# Mild phenotype of knockouts of the major apurinic/apyrimidinic endonuclease APEX1 in a non-cancer human cell line

**DOI:** 10.1371/journal.pone.0257473

**Published:** 2021-09-16

**Authors:** Daria V. Kim, Liliya M. Kulishova, Natalia A. Torgasheva, Vasily S. Melentyev, Grigory L. Dianov, Sergey P. Medvedev, Suren M. Zakian, Dmitry O. Zharkov

**Affiliations:** 1 Department of Natural Sciences, Novosibirsk State University, Novosibirsk, Russia; 2 SB RAS Institute of Chemical Biology and Fundamental Medicine, Novosibirsk, Russia; 3 SB RAS Institute of Cytology and Genetics, Novosibirsk, Russia; 4 Department of Oncology, MRC Oxford Institute for Radiation Oncology, University of Oxford, Oxford, United Kingdom; University of South Alabama Mitchell Cancer Institute, UNITED STATES

## Abstract

The major human apurinic/apyrimidinic (AP) site endonuclease, APEX1, is a central player in the base excision DNA repair (BER) pathway and has a role in the regulation of DNA binding by transcription factors. In vertebrates, APEX1 knockouts are embryonic lethal, and only a handful of knockout cell lines are known. To facilitate studies of multiple functions of this protein in human cells, we have used the CRISPR/Cas9 system to knock out the *APEX1* gene in a widely used non-cancer hypotriploid HEK 293FT cell line. Two stable knockout lines were obtained, one carrying two single-base deletion alleles and one single-base insertion allele in exon 3, another homozygous in the single-base insertion allele. Both mutations cause a frameshift that leads to premature translation termination before the start of the protein’s catalytic domain. Both cell lines totally lacked the APEX1 protein and AP site-cleaving activity, and showed significantly lower levels of the *APEX1* transcript. The APEX1-null cells were unable to support BER on uracil- or AP site-containing substrates. Phenotypically, they showed a moderately increased sensitivity to methyl methanesulfonate (MMS; ~2-fold lower EC_50_ compared with wild-type cells), and their background level of natural AP sites detected by the aldehyde-reactive probe was elevated ~1.5–2-fold. However, the knockout lines retained a nearly wild-type sensitivity to oxidizing agents hydrogen peroxide and potassium bromate. Interestingly, despite the increased MMS cytotoxicity, we observed no additional increase in AP sites in knockout cells upon MMS treatment, which could indicate their conversion into more toxic products in the absence of repair. Overall, the relatively mild cell phenotype in the absence of APEX1-dependent BER suggests that mammalian cells possess mechanisms of tolerance or alternative repair of AP sites. The knockout derivatives of the extensively characterized HEK 293FT cell line may provide a valuable tool for studies of APEX1 in DNA repair and beyond.

## Introduction

Many endogenous and environmental factors generate a steady stream of lesions in cellular DNA. Estimates based on known rates of spontaneous DNA damage suggest that ~20,000 endogenous lesions appear in each cell’s DNA per day [1]. Base excision repair (BER) is the system primarily responsible for the removal of small non-bulky lesions from DNA [2, 3]. BER is initiated by various DNA glycosylases, each of which is specific to a characteristic type of damage. DNA glycosylases recognize damaged bases and hydrolyze their *N*-glycosidic bonds, leaving a baseless deoxyribose residue (apurinic/apyrimidinic site, or AP site). The AP site is further processed by an AP endonuclease (APEX1 in mammals), which cleaves the phosphodiester bond 5′ to the lesion. DNA polymerase β (POLβ) then eliminates the blocking 2′-deoxyribose-5′-phosphate and inserts an undamaged dNMP. At the final stage, the complex of XRCC1 adaptor protein and DNA ligase IIIα seals the remaining nick.

Besides being an intermediate in BER, AP sites are among the most abundant spontaneously arising DNA lesions [4–6]. AP sites are non-instructional and thus strongly mutagenic, often directing dAMP misincorporation [7, 8]. In addition, AP sites are toxic for cells via multiple mechanisms, including DNA strand breakage, transcription errors, and covalent trapping of DNA-bound proteins such as histones and topoisomerases [9–12]. In yeast, a full depletion of the AP site repair capability is lethal [13]. *E*. *coli* cells devoid of AP site-processing activities are exceedingly sensitive to a variety of genotoxic agents and chronically induces the SOS response, indicating persisting DNA damage [14–16].

APEX1 (also known as APE1, HAP1, or Ref-1) is one of the key BER elements involved in the repair of AP sites in human cells [17–20]. In addition to its endonuclease activity, APEX1 exhibits redox activity directed to several transcription factors, such as AP-1 (Jun/Fos), NF-κB, HIF-1a, CREB, and p53 [18, 19, 21, 22], reducing oxidized cysteines in the DNA-binding domains of these proteins and thereby stimulating their ability to bind DNA. Recently, APEX1 was shown to play a role in RNA processing and quality control [23, 24].

To elucidate the effect of AP sites and the role of APEX1 at the cell and organism level, several attempts have been made to obtain knockout mice and cell lines. However, *Apex1*^−/−^ mouse embryos die at the early stages of development and failed to produce cell lines [25–28]. Embryonic fibroblasts can be isolated from transgenic mice with the floxed human *APEX1* gene but enter apoptosis after the induction of the cassette removal [28]. In zebrafish embryos, morpholino knockdown of *apex1* is lethal due to abnormal formation of the early cardiovascular system [29]. On the other hand, *Apex1*^+/−^ mice are viable and fertile but tumor-prone and hypersensitive to oxidative stress [27, 30, 31].

Presently, there are few mammalian cell models with stably inactivated genes encoding APEX1 homologs [32]. A knockout was generated by a conventional recombination approach in the hypotriploid mouse B-cell line CH12F3, producing cells hypersensitive to MMS [33]. Also, a CRISPR/Cas9 knockout derivative of breast cancer HCC1937 cell line was reported [34]. However, the majority of studies on the functional deficiency of APEX1 in mammalian cells so far involved downregulation of *APEX1* expression. Knockdown of *APEX1* sensitizes cells to a wide range of genotoxic assaults, such as ionizing radiation, oxidative stress (H_2_O_2_, paraquat, bleomycin), alkylating agents (methyl methanesulfonate (MMS), temozolomide, thiotepa), crosslinkers, and topoisomerase inhibitors [35–38]. Although the knockdown models proved very useful, to follow the effects of the full lack of APEX1 in the long term it would be desirable to have an APEX1-deficient derivative of a well-characterized cell line. Here we report the generation and characterization of a CRISPR/Cas9 *APEX1* knockout in the extensively studied non-cancer HEK 293FT human cell line. Although these cells were not able to complete BER at the biochemical level and demonstrated an increased level of AP sites, the phenotypic consequences were surprisingly mild, suggesting the existence of AP sites tolerance or alternative repair pathways in mammalian cells.

## Materials and methods

### Cells, plasmids, oligonucleotides and enzymes

HEK 293FT cell line was from Thermo Fisher Scientific (Waltham, MA; Cat. #R70007). The supported laboratory stock was checked by PCR for the absence of mycoplasma contamination. Plasmid pSpCas9(BB)-2A-GFP (PX458; [39]) was a gift from Feng Zhang (Addgene plasmid #48138; http://n2t.net/addgene:48138; RRID: Addgene_48138). Restriction endonucleases AsiGI, DraIII, PctI, proteinase K, and *E*. *coli* uracil–DNA glycosylase (Ung) were purchased from Sibenzyme (Novosibirsk, Russia), restriction endonuclease BbsI and phage T4 DNA ligase were from New England Biolabs (Beverly, MA), and *Taq* DNA polymerase, from Thermo Fisher Scientific (Waltham, MA). Recombinant human APEX1 was purified as described [40]. Recombinant rat DNA POLβ, purified as described [41], was a kind gift from Dr. Nina Moor (SB RAS ICBFM). Polyclonal rabbit anti-APEX1 antibodies raised against the full-length affinity-purified protein (NB100-101) were from Novus Biologicals (Centennial, CO), and secondary goat anti-rabbit antibodies (ab6791) were from Abcam (Cambridge, UK). The oligonucleotides used in this study are listed in S1 Table. The oligonucleotides, including modified ones, were synthesized at the SB RAS ICBFM oligonucleotide synthesis facility and gel-purified before use.

### Generation of knockout lines

Inserts corresponding to the designed sgRNA were cloned into the BbsI site of pX458. This plasmid carries a constitutively expressed *Streptococcus pyogenes* Cas9 gene linked to the eGFP reporter through a T2A ribosome skipping sequence, and a cassette for U6 promoter-driven sgRNA synthesis [39]. The plasmids were purified from *E*. *coli* DH5α using a Plasmid Mini Kit (Qiagen, Venlo, Netherlands), and the correctness of the insert was confirmed by Sanger sequencing. HEK 293FT cells (~10^5^ cells) were transfected with 1.25 μg of the plasmid using Lipofectamine 3000 (Thermo Fisher Scientific). After 48 h, ~1000 GFP-positive cells were collected using S3e Cell Sorter (Bio-Rad Laboratories, Hercules, CA), diluted into 96-well plates at 0.5, 1, and 2 cells per well, and grown as described [39]. The wells containing microscopically observed monoclones were allowed to grow until ~60% monolayer density, after which half of the cells were separated and frozen to maintain the clone. The other half was grown to a 100% monolayer, trypsinized and analyzed. Genomic DNA was obtained by lysis in the buffer containing 10 mM Tris–HCl (pH 8.0), 10 mM EDTA, 3.5% Igepal CA630, 3.5% Tween 20, and 0.4 mg/ml Proteinase K for 3 h at 65°C followed by phenol–chloroform extraction. For tracking of indels by decomposition (TIDE) analysis, the genomic DNA was PCR-amplified, purified with QIAquick PCR Purification Kit (Qiagen) and subjected to Sanger sequencing. For genotyping, the PCR products were digested with DraIII for 1 h at 37°C. For sequencing individual alleles, the PCR products were subcloned into the pCR2.1 vector using TA Cloning Kit (Thermo Fisher Scientific).

### Real-time RT-PCR

Total RNA was purified using RNeasy Plus Mini Kit (Qiagen). Reverse transcription from a (dT)_16_ primer and 1 μg RNA was done with M-MuLV–RH First Strand cDNA Synthesis Kit (Biolabmix, Novosibirsk, Russia) according to the manufacturer’s instructions. PCR was performed on a LightCycler 96 instrument (Roche Applied Science, Penzberg, Germany) using a BioMaster HS-qPCR SYBR Blue kit (Biolabmix). The sequences of the primers are given in S1 Table; all primer pairs were designed to anneal within sequences from different exons or to span an exon/exon border in cDNA. β2-Microglobulin was used as a reference gene to normalize the target gene expression [42]. Two biological replicates were performed, for each one 1–3 independent cDNA preparations were made on different days, each preparation was analyzed in triplicate.

### Enzyme activity in cell extracts

Whole-cell extracts were prepared according to [43]. In the assay for AP endonuclease activity, the reaction mixture (10 μl) included 20 mM Tris–HCl (pH 8.0), 50 mM NaCl, 10 mM MgCl_2_, 2 mM dithiothreitol (DTT), 5% glycerol, 40 ng of the cell extract, and 50 nM 23-mer 5′-fluorescein-labeled duplex substrate containing a (3-hydroxytetrahydrofuran-2-yl)methyl phosphate residue (THF). The reaction was allowed to proceed for 10 min at 37°C and terminated by adding 5 μl of deionized formamide and heating for 5 min at 95°C. The reaction products were analyzed by electrophoresis in 20% polyacrylamide gel containing 8 M urea and visualized using a Typhoon FLA 9500 Imager (GE Healthcare, Chicago, IL). In the assay for uracil–DNA glycosylase activity, the substrate contained dU instead of THF, and the reaction was stopped by adding NaOH to 0.1 M and heating for 2 min at 95°C. The mixture was neutralized by equimolar HCl and processed as above. In the gap-filling assay, the substrate consisted of an 11-mer primer, 40-mer template, and a 28-mer downstream strand (S1 Table), which upon assembly produced a single-nucleotide gap with T in the template. The reaction was performed in 50 mM Tris–HCl (pH 7.5), 10 mM MgCl_2_, 10 mM DTT, 0.1 mM dATP, supplemented with 50 nM substrate and 1 μg cell extract for 30 min at 37°C and processed as indicated above for the AP endonuclease reaction.

### DNA repair assay

The reaction mixture (10 μl) included 50 nM 5′-^32^P-labeled THF:C or U:C substrate and 1 μg cell extract in BER buffer containing 50 mM Tris–HCl (pH 7.5), 10 mM MgCl_2_, 10 mM DTT, 0.1 mM dGTP, and 1 mM ATP, or in POLβ buffer containing 10 mM Tris–HCl (pH 7.6), 10 mM MgCl_2_, 1 mM DTT, and 0.1 mM dGTP. The reaction was allowed to proceed for 30 min at 37°C and terminated by adding 5 μl of formamide dye (20 mM EDTA, 0.1% xylene cyanol, 0.1% bromophenol blue in formamide) and heating for 5 min at 95°C. In the case of the U:C substrate, some of the completed reactions were treated with 100 nM *E*. *coli* Fpg protein for 5 min at 37°C to confirm that the AP site was present, and then terminated as above. The reaction products were analyzed by electrophoresis in 20% polyacrylamide gel containing 8 M urea and visualized using a Typhoon FLA 9500 Imager.

### Doubling time experiments

Cells were grown in Dulbecco’s modified Eagle’s medium supplemented with 10% fetal bovine serum, 100 U/ml penicillin, 100 U/ml streptomycin, and 0.25 μg/ml amphotericin B at 37°C under 5% CO_2_. Exponentially growing cells were seeded in 24-well plates at 4×10^4^/well. Each 24 h the cells were trypsinized and counted using a Luna II automated cell counter (Logos Biosystems, South Korea).

### Cell cycle experiments

Exponentially growing cells were seeded in 6-well plates at 10^4^/well. Twenty-four hours later the cells were collected in cold PBS and counted. One million cells were fixed in 70% ethanol at 4°C for 24 h, washed in PBS and stained in 1 ml of the solution containing 0.1% (v/v) Triton X-100, 20 μg of propidium iodide, 200 μg of DNase-free RNase A for 15 min at 37°C. Immediately after staining, the cells were analyzed using NovoCyte 3000 flow cytometer (Agilent Technologies, Santa Clara, CA); PE Texas Red channel was used to detect the propidium iodide signal.

### Cell viability

The cell viability was determined using 3-(4,5-dimethylthiazol-2-yl)-2,5-diphenyltetrazolium bromide (MTT). Solutions of H_2_O_2_, KBrO_3_, and MMS were prepared in serum-free medium. Cells were seeded in 96-well plates at 4.5×10^3^/well. After 24 h, an equal volume of chemical-containing medium was added to the cells for 24 h (H_2_O_2_) or 48 h (KBrO_3_ and MMS). After the incubation, the growing media was removed, and 0.25 mg/ml MTT solution in the medium was added for 2 h. Then the MTT-containing medium was aspirated, and formazan crystals were dissolved in DMSO. The optical density was measured at 570 nm and 620 nm using Apollo-8 Microplate Absorbance Reader LB 912 (Berthold Technologies, Bad Wildbad, Germany). The cell viability was calculated as the percentage of staining intensity in treated wells relative to the control.

### Aldehyde reactive probe assay

The levels of AP sites were determined using the DNA damage assay kit (ab211154, Abcam, Cambridge, UK) according to the manufacturer’s protocol. Cells were seeded in 6-well plates at 3×10^5^/well. After 24 h, the cells were briefly rinsed in phosphate-buffered saline (PBS) and incubated for 1 h in 1 mM MMS dissolved in dimethyl sulfoxide (DMSO); as a control, 0.1% DMSO solution in PBS was used. Then the cells were again rinsed in PBS and incubated for 2 hours in the complete medium. After that, the cells were collected by trypsinization, washed in PBS, and the genomic DNA was extracted using QIAamp DNA mini kit (Qiagen) and used for AP site determination.

## Results

### *APEX1* gene knockout in HEK 293FT cells

**Fig 1:**
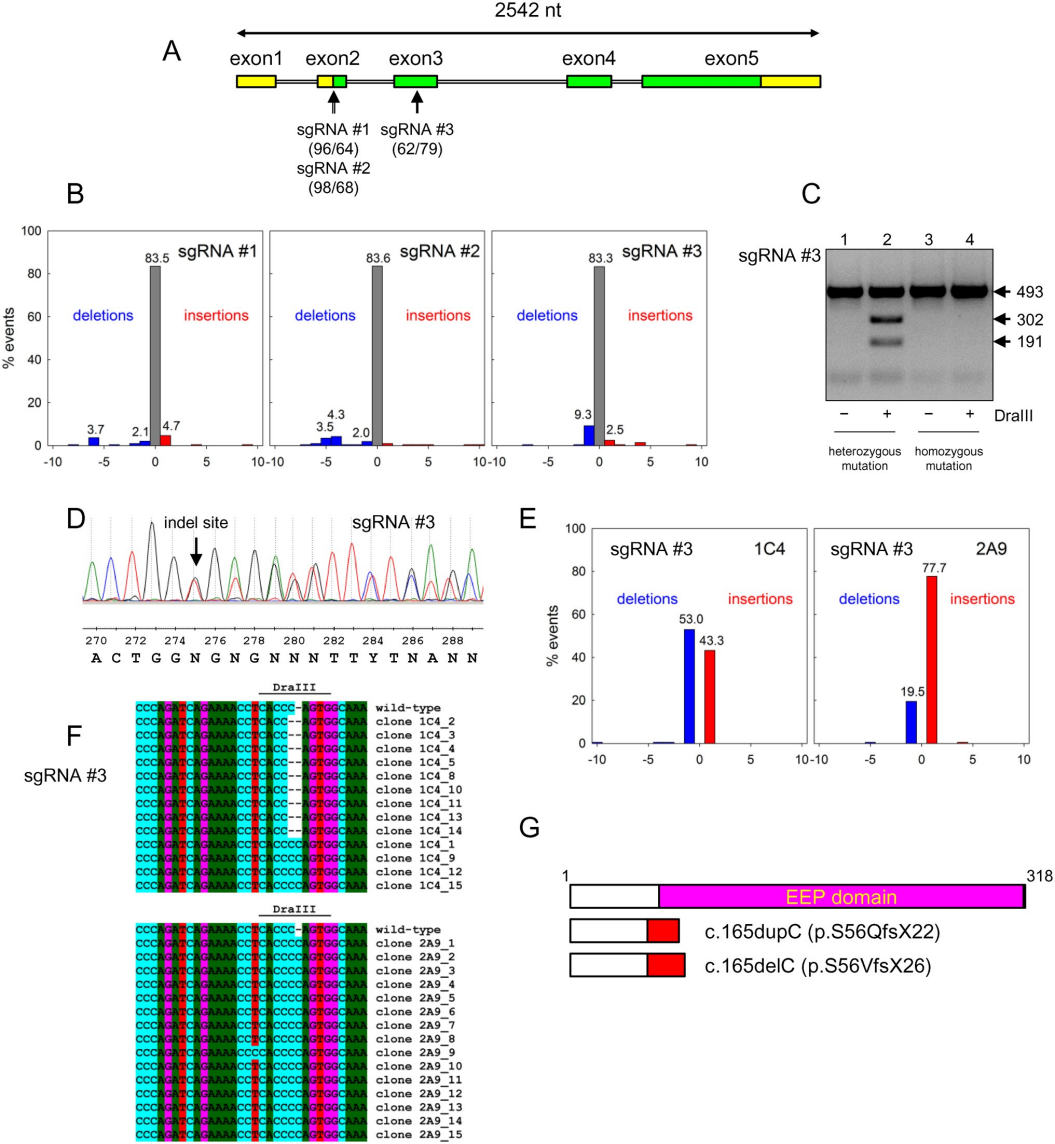
Generation of APEX1 knockout cells using CRISPR/Cas9 technology. **A,** map of human APEX1 gene and location of the protospacers targeted by sgRNAs. Numbers in parentheses correspond to efficiency and specificity scores calculated by benchling.com; higher scores indicate better efficiency and better specificity. Green parts of the exons correspond to the protein-coding sequence. **B,** TIDE analysis of the distribution of mutation events in the pool of HEK 293FT cells after transfection with pX458 expressing different sgRNAs. The blue bars represent deletions, the red bars, insertions, the grey bars, sequences with no mutations. The X axis shows the distance from the target cleavage site in nucleotides. **C,** cleavage of the PCR-amplified part of the *APEX1* gene by DraIII in wild-type cells (*lanes 1*, *2*) and after a successful knockout induced by sgRNA#3 (*lanes 3*, *4*). Arrows mark the mobility of the full-length PCR product (493 nt) and the cleavage fragments. **D,** representative capillary sequencing chromatogram (ABI 3130xl instrument; complementary strand sequenced) of the PCR-amplified part of the *APEX1* gene from the DraIII-negative clone 1C4. **E,** TIDE analysis of the distribution of mutation events in 1C4 and 2A9 single cell clones induced by sgRNA#3. **F,** sequences of the protospacer region in subcloned fragments from 1C4 and 2A9 cells. **G,** truncated APEX1 proteins arising from the single-base insertion and single-base deletion at nucleotide 165. Changed polypeptide parts after the frameshift are colored red. The catalytic EEP domain is shown in magenta.

Using the on-target and off-target effect estimator implemented in the benchling.com platform [44, 45], we have designed three sgRNA sequences targeting protospacers in the human *APEX1* gene, aiming to introduce a frameshift mutation near the beginning of the coding sequence where it would more likely to inactivate the protein product (Fig 1A, S1 Fig). Two of the protospacers were located in exon 2 spanning the initiator ATG codon and were shifted by a single base relative to each other, leading to the use of different protospacer-adjacent motif (PAM) sequences: TGG for Protospacer 1 and GGG for Protospacer 2. The third protospacer was fully located in the coding part of the gene (exon 3).

After the plasmid transfection and sorting of GFP-positive cells, we have amplified the target genomic region by PCR and used tracking of indels by decomposition (TIDE) approach to compare the efficiency of the sgRNA in inducing frameshift mutations [46]. Overall, ~17% of the PCR products carried an insertion or a deletion in all three cases (Fig 1B). However, the products of transfection with a plasmid producing sgRNA targeting Protospacer 3 were mostly single-base deletions or insertions, whereas the other two sgRNAs induced a more diverse range of indels (Fig 1B). Since ±1 indels unambiguously produce a frameshift, we went forward with the cell population resulting from Cas9 targeting by sgRNA #3.

Of 113 obtained monoclones after transfection with pX458 expressing sgRNA#3, we have genotyped 19 random clones using DraIII restriction endonuclease, the site of which (5′-CACNNNGTG-3′) overlaps the 3′-terminus of the protospacer (Fig 1C, S1 Fig). Six of the clones had lost all copies of the DraIII site; two of the clones (1C4 and 2A9) were randomly selected and analyzed by TIDE (Fig 1D and 1E, S2 Fig). In both clones, more than 97% PCR products carried a mutation. In 1C4, we have observed an approximately equal ratio of single-base insertions and deletions (~1.2-fold more deletions), whereas the 2A9 clone carried ~4-fold more +1 insertions than −1 deletions (Fig 1E). To characterize the genotype of the clones more precisely, we have subcloned the PCR products and sequenced 13 clones for 1C4 and 15 clones for 2A9 (Fig 1F). Four of the 1C4 clones carried an insertion of C within the DraIII site (c.165dupC), whereas nine carried a deletion of C within this site (c.165delC). All 2A9 clones carried the c.165dupC insertion, and one of the clones had an additional T>C substitution immediately before the DraIII site (c.160T>C). Since HEK 293FT is a hypotriploid cell line and carries three copies of the 14q11.2 chromosomal region where the *APEX1* gene is located, we conclude that the most likely genotype of 1C4 cells is two c.165delC alleles and one c.165dupC allele, whereas 2A9 carries three c.165dupC alleles, one of which possibly carries a c.160T>C mutation. The presence of this substitution might be a source of erroneous decomposition as a deletion allele by TIDE. Given the number of sequenced subclones, the probability of missing a wild-type allele in monoclones of hypotriploid cells would be ~5×10^−3^ for 1C4 and ~2×10^−3^ for 2A9.

At the polypeptide level, the c.165dupC variant retains the first 55 amino acid residues of the wild-type protein, and, after the frameshift, produces a stop codon, terminating after residue 76 (p.S56QfsX22). The c.165delC variant retains the same first 55 amino acid residues and produces a stop codon after residue 80 (p.S56VfsX26). Both mutations completely eliminate the catalytic domain of the APEX1 protein as well as the Cys65 residue critical for its redox function (Fig 1G, S3 Fig).

### *APEX1* knockout cells lack functional base excision repair

**Fig 2:**
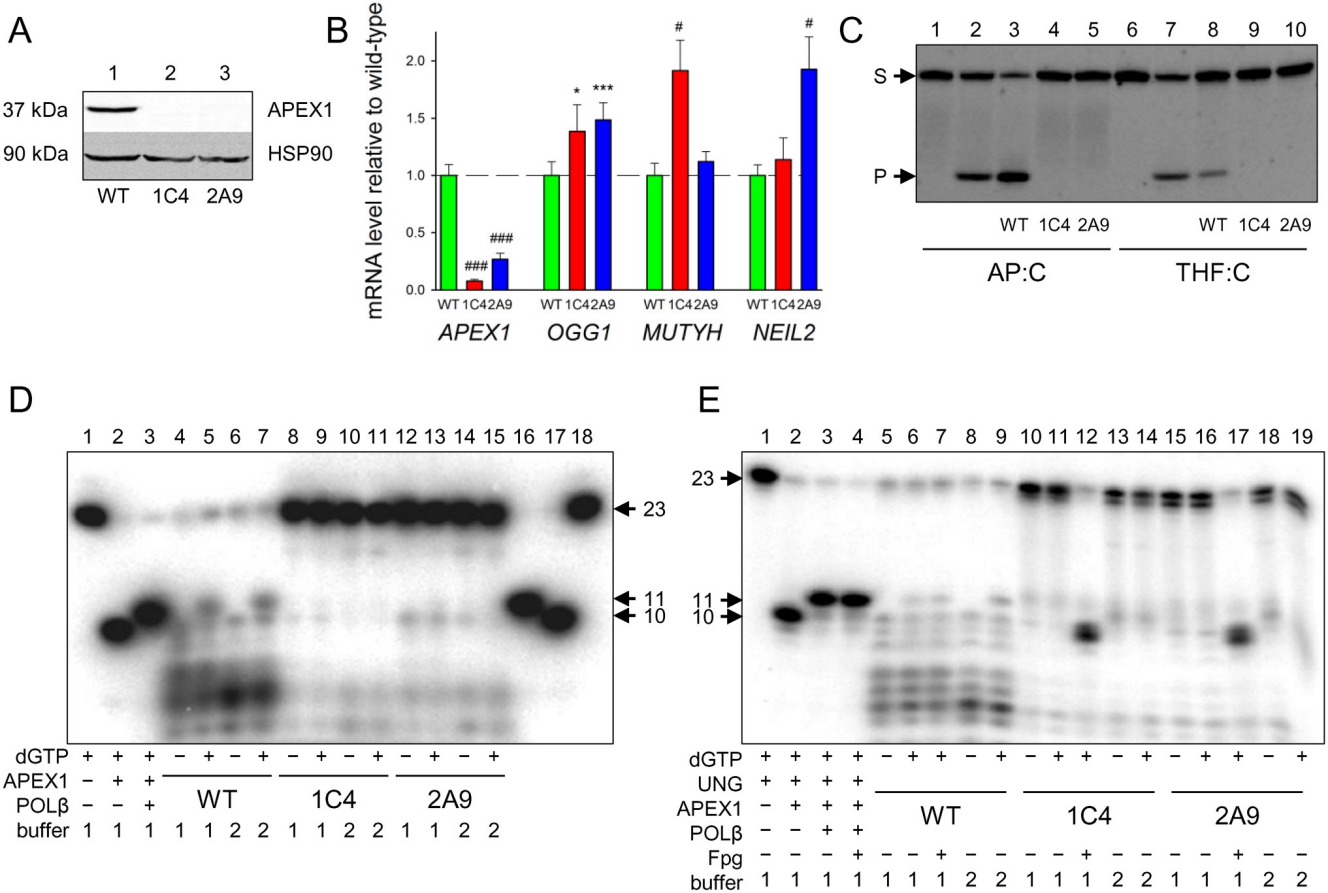
APEX1 knockout cells lack detectable APEX1 protein, AP endonuclease activity, and BER. **A,** Western blot for APEX1 in the extracts of wild-type HEK 293 (*lane 1*) and knockout cells 1C4 (*lane 2*) and 2A9 (*lane 3*). **B,** mRNA levels of *APEX1*, *OGG1*, *MUTYH*, and *NEIL2* genes in 1C4 and 2A9 cells relative to wild-type HEK 293FT. Mean ± s.d. are shown (*n* = 4, see Materials and methods for a detailed description). Student’s unpaired *t* test: *, *p* < 0.05; ***, *p* < 0.005; #, *p* < 0.001; ###, *p* < 0.0001. **C,** cleavage of a 23-mer duplex oligonucleotides containing an AP:C pair (*lanes 1–5*) or a THF:C pair (*lanes 6–10*) by extracts of wild-type HEK 293 (*lanes 3 and 8*) and knockout cells 1C4 (*lanes 4 and 9*) and 2A9 (*lanes 5 and 10*). *Lanes 1 and 6*, no enzyme or cell extract; *lanes 2 and 7*, recombinant APEX1. Arrows mark the mobility of the substrate (S) and the cleavage product (P). **D,** repair of a THF:C duplex. *Lanes 1–3*, repair system reconstituted from purified APEX1 and POLβ, as indicated below the gel. *Lanes 4–7*, extracts of wild-type HEK 293; *lanes 8–11*, extracts of 1C4 cells; *lanes 12–15*, extracts of 2A9 cells. Buffer 1, BER buffer, buffer 2, POLβ buffer (see Materials and methods). *Lanes 16–18*, size markers, as specified by the arrows. **E,** repair of a U:C duplex. *Lanes 1–4*, repair system reconstituted from purified UNG, APEX1 and POLβ, as indicated below the gel. *Lanes 5–9*, extracts of wild-type HEK 293; *lanes 10–14*, extracts of 1C4 cells; *lanes 15–19*, extracts of 2A9 cells. Buffers are as in *Panel D*. Some reactions were post-treated by Fpg to confirm the AP site formation. C–E, representative gels from 3–4 experiments.

To evaluate the consequences of APEX1 deletion in HEK 293FT cells, we have looked at the production of the protein by Western blotting (Fig 2A). Both 1C4 and 2A9 cells lacked the band detectable by anti-APEX1 antibodies, which was clearly seen in wild-type cells. The *APEX1* mRNA level was ~13-fold lower in 1C4 cells and 4-fold lower in 2A9 cells relative to wild-type HEK 293FT, suggesting that the mutant mRNAs undergo nonsense-mediated RNA decay (Fig 2B). In comparison, the levels of mRNA of several DNA glycosylase genes were unaffected or even elevated (Fig 2B, S4 Fig). Notably, we observed a statistically significant increase in *OGG1* mRNA in both knockout lines, whereas *MUTYH* expression was enhanced in 1C4 cells, and *NEIL2* expression, in 2A9 cells (Fig 2B). This may suggest that permanent loss of APEX1 induces a compensatory increase in DNA glycosylases that have an AP lyase activity, or those that participate in the repair of oxidative DNA damage.

We then inquired whether *APEX1* knockout is sufficient to eliminate AP site cleavage activity from the cells. Wild-type cell extracts efficiently cleaved double-stranded oligonucleotides containing an aldehydic AP site or its non-aldehydic analog, (3-hydroxytetrahydrofuran-2-yl)methyl phosphate (THF) (Fig 2C, lanes 3 and 8). However, no cleavage products were observed in the extracts of the knockout cell lines (Fig 2C, lanes 4–5 and 9–10). At the same time, both wild-type and *APEX1*-null cells were fully proficient in the ability to excise uracil from DNA (S5A Fig) and to incorporate a dNMP into a gapped DNA substrate (S5B Fig). This indicates that the BER pathway is functional in the knockout cells both upstream (base removal) and downstream (gap filling) of the AP endonuclease step.

To evaluate the BER proficiency of the knockout cells, we have treated duplexes containing either U or THF residues with the cell extracts in the presence of dNTPs. In the wild-type cell extracts, we observed substrate cleavage followed by strong 3′→5′ exonucleolytic degradation, which may be ascribed to either APEX1 itself or other exonucleases present in the extract. Interestingly, such degradation was not apparent in a gapped substrate in the polymerase experiments (S5B Fig), suggesting a requirement for an assembly of a full BER complex. Despite the exonuclease degradation, insertion of a dNTP after APEX1 cleavage was evident by the appearance of the 11-mer product (Fig 2D, lanes 5 and 7, Fig 2E, lanes 6, 7, and 9). In an attempt to enhance the accumulation of the 11-mer, we have used the reaction mixture optimal for POLβ-catalyzed gap filling [47], yet essentially the same results were observed as in the BER buffer (compare lanes 4–5 and 6–7 in Fig 2D and 5–6 and 8–9 in Fig 2E). Contrary to the wild-type extracts, only minor substrate cleavage was observed in both knockout lines, even though uracil was efficiently removed, as evidenced by the efficient cleavage of the reaction product by Fpg, a DNA glycosylase with a strong AP lyase activity (Fig 2D, lanes 8–15, Fig 2E, lanes 10–19). Cleavage products present in trace amounts in the reactions with the U:C substrate have lower mobility than the APEX1 product and likely reflect β-elimination of aldehydic AP sites by any of AP lyases present in the cell, which generate 3′-unsaturated aldehydes that cannot be extended by DNA polymerases (S6 Fig).

### Biological consequences of *APEX1* knockout

**Fig 3:**
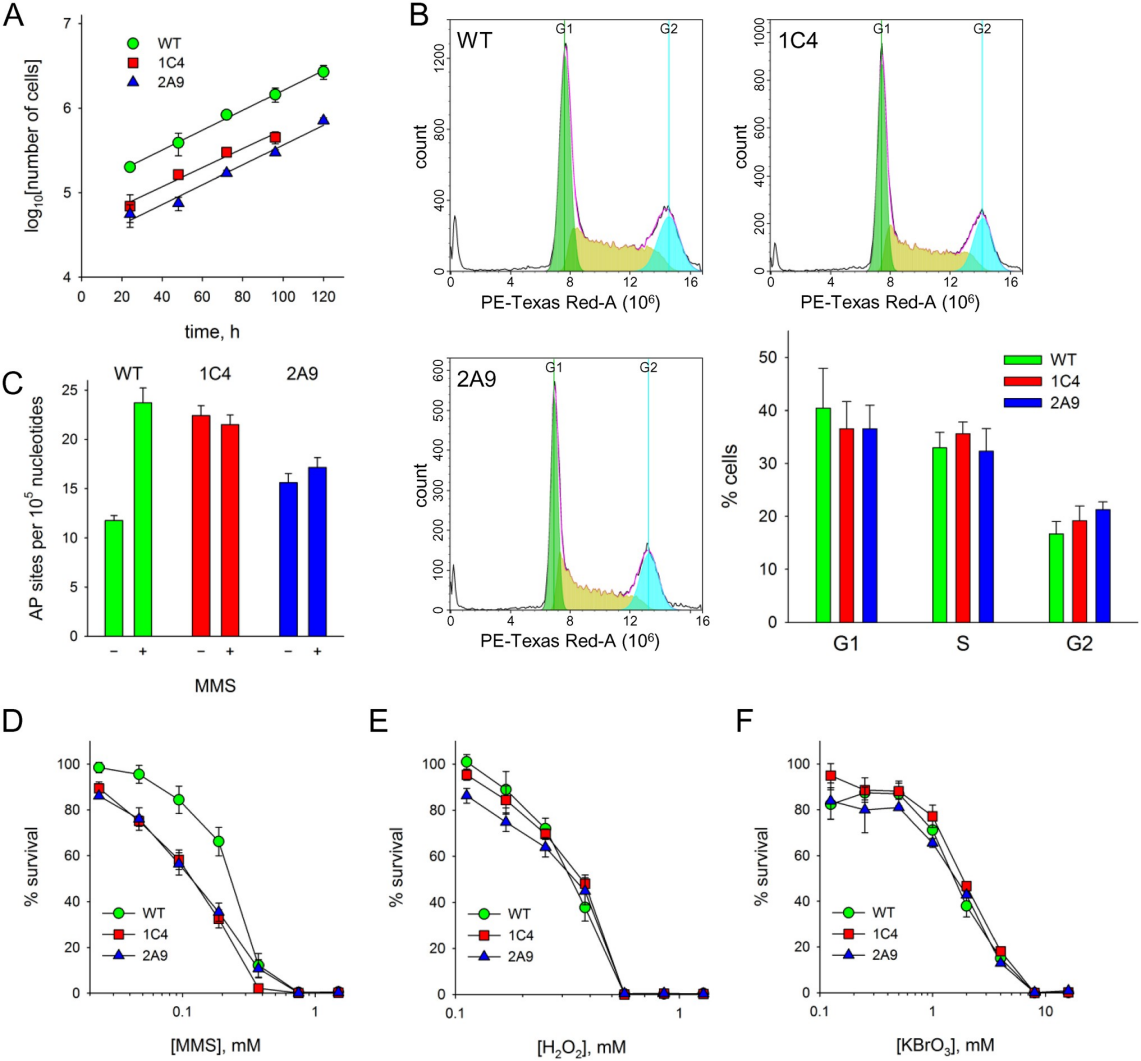
Phenotypic consequences of the APEX1 knockout. **A,** growth curves of wild-type HEK 293 (*circles*) and knockout cells 1C4 (*squares*) and 2A9 (*triangles*). Mean ± s.d. is shown (*n* = 2). **B,** cell cycle analysis of wild-type and knockout cells using propidium iodide staining. Representative cell count histograms and percentage of cells in G1, S and G2 phases in a non-synchronized population (*n* = 3, mean ± s.d.) are shown. **C,** accumulation of AP sites in wild-type HEK 293 (*green*) and knockout cells 1C4 (*red*) and 2A9 (*blue*) with and without MMS treatment. Mean ± s.d. is shown (*n* = 2). **D–F,** sensitivity of wild-type HEK 293 and knockout 1C4 and 2A9 cells to MMS (*Panel D*, *n* = 4), H_2_O_2_ (*Panel E*, *n* = 3) and KBrO_3_ (*Panel F*, *n* = 3). Cell line symbols are as in *Panel A*. Mean ± s.d. is shown.

Having confirmed that the *APEX1* knockout lines are unable to support BER at the biochemical level, we set out to explore how this deficiency is manifested in the cells’ phenotype. The mutant cells showed no obvious deviations in the overall cell morphology. Wild type and knockout cells showed no significant difference in population doubling time (21.7 h HEK 293FT, 20.4 h 1C4, 19.4 h 2A9, Fig 3A) or in the percentage of cells at different cell cycle stages (Fig 3B).

Knockout of *APEX1* would be expected to increase the abundance of background AP sites in the genomic DNA. To see if this is indeed the case, we have quantified the AP sites using the aldehyde-reactive probe (ARP) assay, which is based on the specific condensation of alkoxyamines with the open form of AP sites [48]. Knockout of *APEX1* led to a ~1.5–2-fold increase in the number of AP sites, comparable with the increase seen in wild-type cells in the presence of MMS (Fig 3C, S7 Fig). Interestingly, when the knockout cells were treated by MMS, we observed no additional increase in the AP sites abundance (Fig 3C). The expression of *MPG* in the *APEX1* null lines tended to be lower but this decrease did not reach statistical significance (S4 Fig), so it is unlikely that extra AP sites do not appear due to the lack of adduct excision by the glycosylase. BER-induced breaks at AP sites are believed to be the main MMS-induced cytotoxic lesions [49], so it is possible that in the absence of APEX1 the reactive aldehydic AP sites are channeled into more deleterious lesions undetectable by ARP, such as DNA–protein cross-links or oxidized AP sites [50, 51].

DNA repair-deficient cells are often characterized by increased sensitivity to various genotoxic agents. We assayed the viability of *APEX1* null lines in the presence of a range of concentrations of hydrogen peroxide, potassium bromate, and methyl methanesulfonate. H_2_O_2_ produces a wide range of lesions in DNA, including single-strand breaks and many oxidized bases [52, 53]. KBrO_3_ predominantly produces 8-oxoguanine and strand breaks in a glutathione-dependent manner [54, 55]. In contrast, MMS mostly methylates guanines at N7 and adenines at N3 [56]. The resulting ring-alkylated purines are quickly converted to AP sites either by methylpurine–DNA glycosylase MPG or by spontaneous hydrolysis [49, 57, 58]. Both 1C4 and 2A9 cell lines demonstrated moderately enhanced sensitivity to MMS compared with the parent HEK 293FT cells (Fig 3D; EC_50_ ~250 μM for wild-type cells and ~120 μM for both 1C4 and 2A9). However, as can be seen in Fig 3E and 3F, no significant difference was found with respect to the sensitivity of all three cell lines to H_2_O_2_ and KBrO_3_, except for 2A9 cells a 100 μM H_2_O_2_ (*p* < 0.05 from both wild-type and 1C4 cells), indicating the existence of back-up repair pathways for cytotoxic lesions produced by these agents in addition to APEX1-dependent BER.

## Discussion

APEX1 is the first common enzyme in the human BER pathway, required for the repair of all small non-bulky damaged bases and AP sites. While many bacteria have two AP endonucleases of unrelated structure but with overlapping enzyme activities (Xth and Nfo in *E*. *coli*), APEX1 seems to be the major human AP endonuclease supporting most of BER and carrying out additional functions such as stimulation of transcription factors binding to DNA, RNA quality control, and regulation of caspase-independent apoptosis [18, 23, 24, 59, 60]. Due to this plethora of functions and the pivotal position in the BER pathway, attempts to produce transgenic animals devoid of APEX1 have so far been unsuccessful [25–29]. Hemizygous mice are viable but demonstrate reduced BER capacity and elevated spontaneous mutagenesis [25, 27, 30, 31]. They also show perturbed vascular tone regulation due to lowered eNOS activation by SIRT1 deacetylase, which depends on the stimulation of SIRT1 by the redox function of APEX1 [61, 62]. Naturally occurring reduced-activity mutants of human APEX1 are known [63, 64] but are exceedingly rare in the general population as germline-inherited alleles [65] so their contribution to human pathology cannot be assessed reliably.

With the advent of multiplex CRISPR/Cas9 genome editing, genome-wide knockout screens became increasingly popular to uncover the genetic basis of cell phenotypes [66]. In several recent CRISPR screens, *APEX1* knockout was shown to sensitize cells to bleomycin, temozolomide, MMS, KBrO_3_ and illudin S (a fungal DNA-damaging sesquiterpene) [67, 68] and negatively affect cell proliferation [67, 69–72]. However, the uncovered phenotypes very much depend on the particular cell line and do not appear in many other CRISPR screens. For example, in one study only one of six patient-derived glioblastoma cell lines showed sensitivity to temozolomide upon *APEX1* knockout [67]. In the same study, the knockout affected proliferation in one of two primary neural stem cell lines and one of ten glioblastoma lines. Obviously, an *APEX1* knockout in a well-characterized cell line would be a valuable resource to study phenotypes resulting from BER inactivation.

In this study, we have knocked out the *APEX1* gene in the human HEK 293FT line and characterized the resulting cells genetically, biochemically and phenotypically. HEK 293FT is a derivative of HEK 293 line, which was long believed to originate from human embryonic kidney tissue but now, based on transcriptome profiling of several descendant lineages, is considered to come from a developing adrenal gland [73]. The group of HEK 293 descendants is surpassed only by HeLa cells in the number of research papers and only by CHO cells as a biotech production platform, and is extensively characterized at the genomic, transcriptomic and proteomic levels [73, 74]. Although HEK 293T and -FT, due to the integrated adenoviral *E1A*/*E1B* genes and SV40 large T antigen expression, are more resistant to apoptosis than many other human cell lines [75, 76], they preserve intact DNA repair systems, including BER [77, 78].

We have found that the knockout cells are unable to cleave both natural and tetrahydrofuran AP sites and are deficient in BER. Although APEX1 is the major human AP endonuclease, other proteins have also been reported to cleave AP sites in human cells. APEX2, an enzyme that belongs to the same exonuclease–endonuclease–phosphatase (EEP) structural superfamily as APEX1, can hydrolyze DNA 5′ to AP sites, however, this activity is relatively weak compared with its 3′→5′-exonuclease and 3′-phosphodiesterase activities [79, 80]. The same activity was reported for aprataxin and polynucleotide kinase/3′-phosphatase like factor (APLF), a multifunctional DNA repair protein structurally unrelated to APEX1 and APEX2 [81]. Finally, many DNA glycosylases contain an AP lyase activity that efficiently cleaves DNA 3′ of aldehydic AP sites (but not THF) by β-elimination [82, 83]. However, APEX1 is a very abundant protein in human cells, including HEK 293 and its derivatives [74, 84], and all other AP endonucleases and lyases combined are evidently insufficient to support a detectable level of activity in the knockout cells.

In the absence of knockout models, most knowledge about the consequences of APEX1 functional deficiency was obtained from knockdown experiments. As with CRISPR screens mentioned above, the effects of *APEX1* downregulation are considerably cell line-dependent. It also should be kept in mind that some effects seen in APEX1-deficient cells may reflect the absence of redox or RNA-binding function of APEX1 rather than the lack of BER. Regarding HEK 293 cells and their descendants, siRNA knockdown of *APEX1* enhanced the sensitivity of HEK 293T to cisplatin and etoposide and led to a more pronounced G2/M arrest upon cisplatin treatment [85]. This, however, may not reflect BER deficiency and was attributed to a disruption of YB-1–p300 transcription regulator recruitment, which depends on APEX1 acetylation [85]. *APEX1* silencing caused an increase in apoptosis under H_2_O_2_ treatment [86]; however, overall cell survival was not reported in that study. In a TET1-mediated global genome demethylation model in HEK 293T cells, *APEX1* knockdown failed to prevent clearance of 5-methylcytosine from DNA [87], suggesting the existence of some backup repair pathway for epigenetic bases oxidized by TET dioxygenases. Overall, the relatively mild phenotype in our knockout lines is consistent with the observations in HEK cells with *APEX1* downregulation. Notably, the ~2-fold increase in the background AP sites level observed in our HEK-derived cells coincides well with the results of genome-wide single-nucleotide resolution AP-specific sequencing (snAP-seq) in HeLa cells after *APEX1* knockdown, where the amount of spontaneously arising AP sites was elevated ~1.8-fold [6].

Both our knockout cell lines showed moderately lower resistance to MMS and were indistinguishable from wild-type cells in the sensitivity to H_2_O_2_ and KBrO_3_. The last two agents mostly produce oxidative lesions, for which an APEX1-independent, polynucleotide kinase/3′-phosphatase (PNKP) BER branch has been described [88, 89]. In this case, BER is initiated by helix–two-turn–helix superfamily DNA glycosylases, such as NEIL1 or NEIL2, which catalyze β,δ-elimination forming a single-nucleotide gap flanked by two phosphates. PNKP further removes the 3′-phosphate generating a suitable end for a repair DNA polymerase. Also, there is growing evidence that the nucleotide excision repair pathway might contribute to oxidative damage repair in human cells [90]. H_2_O_2_ also yields “dirty” single-strand breaks with oxidized 3′-terminal sugar fragments that need to be removed before repair. APEX1 has this activity but human cells possess several other enzymes that can serve as 3′-repair phosphodiesterases, including APEX2, TDP1, and XPF–ERCC1 [80, 91, 92].

The case of MMS is more intriguing, as we see increased cell death but no accumulation of extra AP sites in the knockout cells. It should be emphasized that our cell survival assay employed 48 h incubation with the drug while AP site measurements were done in an acute exposure mode (1 mM MMS for 1 h), which seems to be optimal for the ARP assay [5, 93, 94]. In the pilot screening of MMS treatment conditions, concentrations >1 mM were poorly tolerated by our cells in an acute mode. The MMS exposure approximately doubled the AP sites number in wild-type cells, consistent with the literature data employing similar treatment regimens [93, 94] but had no effect on the intrinsically higher levels of ARP-detectable AP sites in the knockout cells. This lack of additional AP sites cannot be explained by the absence of excision of methylated bases by MPG, since the expression of *MPG* decreases only marginally. We hypothesize that in APEX1-deficient cells, the AP sites generated by MPG and not immediately cleaved by concerted AP endonuclease action [95] could be eventually converted to dirty strand breaks, oxidized AP sites, or DNA–protein cross-links that contribute to cell death but are hidden from ARP. Also, homologous recombination is known to contribute significantly to the prevention of MMS-induced cytotoxicity in mammalian cells [96] and could serve as a backup repair pathway in APEX1-deficient cells.

## Conclusions

In summary, we have produced two APEX1 null derivatives of the commonly used HEK 293FT human non-cancer cell line and characterized them at the genetic, biochemical and phenotypic level. The knockout nature of the clones is supported by (i) lack of the wild-type allele in individually sequenced subclones; (ii) lack of protein band detectable by anti-APEX1 antibodies in the cell extracts; (iii) lack of THF- and AP site-cleaving activity in cell extracts. Despite the total abolishment of the canonical BER activity, the phenotypes of the knockout lines turned out to be relatively mild, with about a twofold increase in the background AP site levels and MMS sensitivity but nearly wild-type resistance to oxidants such as hydrogen peroxide or potassium bromate. This could indicate that mammalian cells possess mechanisms to tolerate AP sites. Another possibility is that alternative, APEX1-independent subpathways of BER relying on bifunctional DNA glycosylases, while under the limit of detection in our experiments, might still be sufficient to keep the level of AP sites low enough for cell survival. Given the wealth of information about the parent HEK 293FT line, we hope that our knockout lines will prove useful for studies of these DNA repair pathways, their interactions, and non-repair functions of APEX1 in human cells.

## Supporting information

S1 Table Oligonucleotides used in this study. (PDF)

S1 Fig Sequence of the *APEX1* gene. Coding parts of the exons are highlighted green, non-coding parts of the exons are yellow. Protospacers are underlined and shown in bold. The DraIII site is boxed.
(PDF)

S2 Fig Cleavage of the PCR-amplified part of the *APEX1* gene by DraIII. Cleavage in wild-type hypotriploid HEK293FT cells (*lanes 2*, *3*) and in wild-type nearly diploid Burkitt lymphoma BL2 cells (*lanes 4*, *5*) is shown. Arrows mark the mobility of the full-length PCR product (493 nt) and the cleavage fragments. *Lane 1*, 100-bp molecular weight markers; *lane 6*, PCR reaction with no primers.
(PDF)

S3 Fig Sequence of the full-length APEX1 protein and its truncated variants. Sequences arising from a single-base insertion at coding position 165 (p.S56QfsX22) or a single-base deletion at coding position 165 (p.S56VfsX26) are shown. Changed polypeptide parts after the frameshift are highlighted red. The catalytic EEP domain is shown in magenta. The Cys65 residue critical for the Ref-1 activity is highlighted green.
(PDF)

S4 Fig mRNA levels of *UNG*, *TDG*, *SMUG1*, *MBD4*, *NTHL1*, *NEIL1*, *NEIL3*, and *MPG* genes coding for various DNA glycosylases in 1C4 and 2A9 cells relative to wild-type HEK 293FT. Mean ± s.d. are shown (*n* = 2–4; see Materials and methods for a detailed description)). None of the differences between knockout and wild-type cells were statistically significant (Student’s *t* rest).
(PDF)

S5 Fig Uracil–DNA glycosylase and gap-filling activities in cell extracts. **A,** cleavage of a 23-mer duplex oligonucleotides containing an U:C pair by extracts of wild-type HEK293 (*lane 3*) and knockout cells 1C4 (*lane 4*) and 2A9 (*lane 5*). *Lane 1*, no enzyme or cell extract; *lane 2*, recombinant Ung. The reaction mixtures were treated with hot alkali to cleave DNA at AP sites formed by uracil removal. Arrows mark the mobility of the substrate (S) and the cleavage product (P). **B,** primer extension in a gapped substrate by extracts of wild-type HEK293 (*lanes 3–4*) and knockout cells 1C4 (*lanes 5–6*) and 2A9 (*lanes 7–8*). *Lanes 1–2*, recombinant POLβ. Arrows mark the mobility of the primer and the extension product.
(PDF)

S6 Fig Products generated from AP site by AP endonucleases and AP lyases. (PDF)

S7 Fig Calibration curve of the aldehyde reactive probe assay. (PDF)

S1 Raw images Original images of gels and blots. (PDF)
