# Supplementary material for: Mild phenotype of knockouts of the major apurinic/apyrimidinic endonuclease APEX1 in a non-cancer human cell line
Source: PLoS One. 2021 Sep 16;16(9):e0257473. doi: 10.1371/journal.pone.0257473 (PMC8445474; doi:10.1371/journal.pone.0257473)
Supplement: S1 Table — (PDF) [file pone.0257473.s001.pdf]

S1 Table. Oligonucleotides used in this study.

ID	Sequence (5′→3′)
**sgRNA-coding inserts for *APEX1***	
APEX1_1_fwd	CACCGGTAACGGGAATGCCGAAGCG
APEX1_1_rev	AAACCGCTTCGGCATTCCCGTTACC
APEX1_2_fwd	CACCGTAACGGGAATGCCGAAGCGT
APEX1_2_rev	AAACACGCTTCGGCATTCCCGTTAC
APEX1_3_fwd	CACCGGATCAGAAAACCTCACCCAG
APEX1_3_rev	AAACCTGGGTGAGGTTTTCTGATCC
**pX458 sequencing primer**	
Pr_3_rev	CAGGTGCTCCAGGTAATTAAAC
**Primers for mutation screening**	
screen1/2_fwd	AGGATTTAGAGATAACGTGGTTTGA
screen1/2_rev	CCTGCTGCCTCTTTGTCATT
screen3_fwd	GGCTTTCGTTGGGTCTATAGTTA
screen3_rev	CAACATTCTGGGAAGAGGAGAG
**Real-time PCR primers**	
rtAPEX1_fwd	GTTTCTTACGGCATAGGCGAT
rtAPEX1_rev	CACAAACGAGTCAAATTCAGCC
rtMBD4_fwd	CCGTCACCTCTAGTGAGCG
rtMBD4_rev	GCAGAAGCGATGGGTTCTTGTA
rtMPG_fwd	CCCATACCGCAGCATCTATTT
rtMPG_rev	GGCTGGTCGAAGAACTCCAA
rtMUTYH_fwd	ATACCGGATGGATGCAGAAGT
rtMUTYH_rev	GCCCAGAGTTGATTCACCTCC
rtNEIL1_fwd	CCTACCGCATCTCAGCTTCAG
rtNEIL1_rev	GTCCACGAAACATAGGGCGAG
rtNEIL2_fwd	CTGTCTGCTATACACTGCTGGA
rtNEIL2_rev	GCACTCAGGACTGAACCGA
rtNEIL3_fwd	TGGATCAGAACGTATTGCCTGG
rtNEIL3_rev	GACCACAATTAGGACGCTTGTAA
rtNTHL1_fwd	TGAGCACTGCTATGACTCCAG
rtNTHL1_rev	AGCGTGGCATCATCTGTCTG
rtOGG1_fwd	ACTCCCACTTCCAAGAGGTG
rtOGG1_rev	GGATGAGCCGAGGTCCAAAAG
rtSMUG1_fwd	CGCAACTACGTGACTCGCTAC
rtSMUG1_rev	ACTGGTCGTTTAGGATGCTCTT
rtTDG_fwd	TCACACTCTACCAGGGAAGTATG
rtTDG_rev	ACGTCCTCCTTCACGAAATTCT
rtUNG_fwd	CCCCACACCAAGTCTTCACC
rtUNG_rev	TTGAACACTAAAGCAGAGCCC
rtB2M_fwd	ATGTCTCGCTCCGTGGCCTTA
rtB2M_rev	ATCTTGGGCTGTGACAAAGTC
**Enzyme substrates**	
U	CTCTCCCTTC**X**CTCCTTTCCTCT (X = uracil)
THF	CTCTCCCTTC**X**CTCCTTTCCTCT (X = THF)
compl	AGAGGAAAGGAGCGAAGGGAGAG
primer	CGAGACCGTCG
downstream	GAGGAAAGAAGCGAAGGAATTCCAGAGC
template	GCTCTGGAATTCCTTCGCTTCTTTCCTCTCGACGGTCTCG
**Size markers**	
23	CTCTCCCTTCGCTCCTTTCCTCT
11	CTCTCCCTTCG
10	CTCTCCCTTC
