# Supplementary material for: Mild phenotype of knockouts of the major apurinic/apyrimidinic endonuclease APEX1 in a non-cancer human cell line
Source: PLoS One. 2021 Sep 16;16(9):e0257473. doi: 10.1371/journal.pone.0257473 (PMC8445474; doi:10.1371/journal.pone.0257473)
Supplement: S1 Fig — Coding parts of the exons are highlighted green, non-coding parts of the exons are yellow. Protospacers are underlined and shown in bold. The DraIII site is boxed. (PDF) [file pone.0257473.s002.pdf]

>NG_008718.1:5096-7637 Homo sapiens apurinic/apyrimidinic endodeoxyribonuclease 1 (APEX1), RefSeqGene on chromosome 14

CTTCTTTGTGCTCGGGTTAGGAGGAGCTAGGCTGCCATCGGGCCGGTGCAGATACGGGGTTGCTCTTTTGCTCATAA
GAGGGGCTTCGCTGGCAGTCTGAACGGCAAGCTTGAGTCAGGACCCTTAATTAAGATCCTCAATTGGCTGGAGGGCA
GATCTCGCGAGTAGGGTACAAGGCACTATGAAATGATCTAGTTTCGTGGGTGAGGGGCTGAAGGGCCTATGATGCAC
GGAGGCGGGGAAAGGATTTAGAGATAACGTGGTTTGAAAGGCGGGACCTGGTGCGGGGACGCTCTTGGGAGGAGTCT
TCTCCCCAGCCTTAGCTGGTTTCATGATTTCTTTGCGTCTGTAGGCAACGCGGTAAAAATATTGCTTCGGTGGGTGA
CGCGGTACAGCTGCCCAAGGGCGTTC**GTAACGGGAATGCCGAAGCGT**GGGAAAAAGGGAGCGGTGGCGGAAGACGGG
GATGAGCTCAGGACAGGTAAGGGAATGAAATCAGCCCTTCTTCCTAGAAGCTGCGGCGGGGGTGTTTGTCATTCCCT
TGATGTACGGTAAGTACGGGCCGACTCATTTTTGCAGGGGTTTGTGAAGAAGTCGCAGGAACCGTAGGCTTTCGTTG
GGTCTATAGTTAACGCCGGATCGCAGTTGGAAACCACCAGCTTTTTGTCAGTATATATTACTCATTTTATAGAGCCA
GAGGCCAAGAAGAGTAAGACGGCCGCAAAGAAAAATGACAAAGAGGCAGCAGGAGAGGGCCCAGCCCTGTATGAGGA
CCCCCCA**GATCAGAAAACCTCACCCAG**TGGCAAACCTGCCACACTCAAGATCTGCTCTTGGAATGTGGATGGGCTTC
GAGCCTGGATTAAGAAGAAAGGATTAGATGTGAGTGGAATTTGAGGGAAAGAGACATTTTTTAGTATTGAATGGTCT
TAGGGTTTAGTCACCCCTTTTCTCCGTTTAGCCTTCAGGCTGTTTTATTTTTCTCCTGCCCGTAGTTTTCTGTGGGG
CTTCCCCAGTCTTGCCAGTTGTATTTCCTAAATGTCTGTTCCTTCACTTCCATTGCCATTTTCTTTTTTAGTGTTCT
CTCCTCTTCCCAGAATGTTGCAAAAACCTCTTCACTATACTTCCTCCATTTTATCTTCCTGCATTGCATTCCATATG
AAGCATGTCCTCCATTCCATTAACCATAGCTTAAAAATCTTAGCTTGCTATCCACTGCCTATAGAAAAAACACATCT
CCTTGGCATAGCATGTAAGACTTTCTTACCTCTCTATATTTGTTTTCATTTATCTAGCTTAGAATTGTTTGAATATT
GTGCTGCTTGACTCGAACTCCTTAGGCCAAGAGACTGTTTAACCCGTGCGTATCTATGACTTAGCATATAGATTATT
CAATAAATGTTCTGCTGAATTGATAATACGTTTTCCACCTTTCTTTTCACTTACAGTGGGTAAAGGAAGAAGCCCCA
GATATACTGTGCCTTCAAGAGACCAAATGTTCAGAGAACAAACTACCAGCTGAACTTCAGGAGCTGCCTGGACTCTC
TCATCAATACTGGTCAGCTCCTTCGGACAAGGAAGGGTACAGTGGCGTGGGCCTGCTTTCCCGCCAGTGCCCACTCA
AAGTTTCTTACGGCATAGGTGAGACCCTATTGATGCCTAATGCCTGAACTCTTCAAAACCAATTGCTAATTCTCTAT
CTCTGCCCCACCTCTTGATTGCTTTCCCTTTTCTTATAGTTTTTTATGCTAATTCTGTTTCATTTCTATAGGCGATG
AGGAGCATGATCAGGAAGGCCGGGTGATTGTGGCTGAATTTGACTCGTTTGTGCTGGTAACAGCATATGTACCTAAT
GCAGGCCGAGGTCTGGTACGACTGGAGTACCGGCAGCGCTGGGATGAAGCCTTTCGCAAGTTCCTGAAGGGCCTGGC
TTCCCGAAAGCCCCTTGTGCTGTGTGGAGACCTCAATGTGGCACATGAAGAAATTGACCTTCGCAACCCCAAGGGGA
ACAAAAAGAATGCTGGCTTCACGCCACAAGAGCGCCAAGGCTTCGGGGAATTACTGCAGGCTGTGCCACTGGCTGAC
AGCTTTAGGCACCTCTACCCCAACACACCCTATGCCTACACCTTTTGGACTTATATGATGAATGCTCGATCCAAGAA
TGTTGGTTGGCGCCTTGATTACTTTTTGTTGTCCCACTCTCTGTTACCTGCATTGTGTGACAGCAAGATCCGTTCCA
AGGCCCTCGGCAGTGATCACTGTCCTATCACCCTATACCTAGCACTGTGACACCACCCCTAAATCACTTTGAGCCTG
GGAAATAAGCCCCCTCAACTACCATTCCTTCTTTAAACACTCTTCAGAGAAATCTGCATTCTATTTCTCATGTATAA
AACTAGGAATCCTCCAACCAGGCTCCTGTGATAGAGTTCTTTTAAGCCCAAGATTTTTTATTTGAGGGTTTTTTGTT
TTTTAAAAAAAAATTGAACAAAGACTACTAATGACTTTGTTTGAATTATCCACATGAAAATAAAGAGCCATAGTTTC
A

S1 Fig. Sequence of the *APEX1* gene. Coding parts of the exons are highlighted green, non-coding parts of the exons are yellow. Protospacers are underlined and shown in bold. The DraIII site is boxed.
