# Supplementary material for: Mild phenotype of knockouts of the major apurinic/apyrimidinic endonuclease APEX1 in a non-cancer human cell line
Source: PLoS One. 2021 Sep 16;16(9):e0257473. doi: 10.1371/journal.pone.0257473 (PMC8445474; doi:10.1371/journal.pone.0257473)
Supplement: S2 Fig — Cleavage in wild-type hypotriploid HEK293FT cells (lanes 2, 3) and in wild-type nearly diploid Burkitt lymphoma BL2 cells (lanes 4, 5) is shown. Arrows mark the mobility of the full-length PCR product (493 nt) and the cleavage fragments. Lane 1, 100-bp molecular weight markers; lane 6, PCR reaction with no primers. (PDF) [file pone.0257473.s003.pdf]

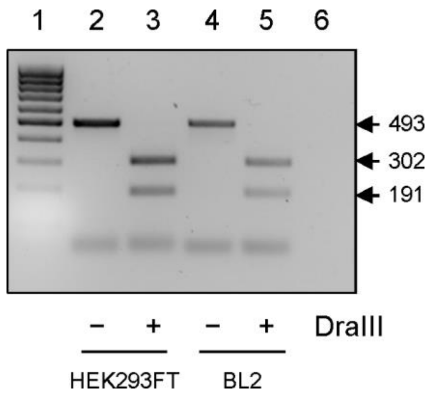

S2 Fig. Cleavage of the PCR-amplified part of the *APEX1* gene by DraIII. Cleavage in wild-type hypotriploid HEK293FT cells (*lanes 2, 3*) and in wild-type nearly diploid Burkitt lymphoma BL2 cells (*lanes 4, 5*) is shown. Arrows mark the mobility of the full-length PCR product (493 nt) and the cleavage fragments. *Lane 1*, 100-bp molecular weight markers; *lane 6*, PCR reaction with no primers.
