# Supplementary material for: Mild phenotype of knockouts of the major apurinic/apyrimidinic endonuclease APEX1 in a non-cancer human cell line
Source: PLoS One. 2021 Sep 16;16(9):e0257473. doi: 10.1371/journal.pone.0257473 (PMC8445474; doi:10.1371/journal.pone.0257473)
Supplement: S3 Fig — Sequences arising from a single-base insertion at coding position 165 (p.S56QfsX22) or a single-base deletion at coding position 165 (p.S56VfsX26) are shown. Changed polypeptide parts after the frameshift are highlighted red. The catalytic EEP domain is shown in magenta. The Cys65 residue critical for the Ref-1 activity is highlighted green. (PDF) [file pone.0257473.s004.pdf]

>NP_001632.2 DNA-(apurinic or apyrimidinic site) endonuclease [Homo sapiens]
MPKRGKKGAVAEDGDELRTEPEAKKSKTAAKKNDKEAAGEGPALYEDPPDQKTSPSGKPATLKICSWNVDGLRAWIK
KKGLDWVKEEAPDILCLQETKCSENKLPAELQELPGLSHQYWSAPSDKEGYSGVGLLSRQCPLKVSYGIGDEEHDQE
GRVIVAEFDSFVLVTAYVPNAGRGLVRLEYRQRWDEAFRKFLKGLASRKPLVLCGDLNVAHEEIDLRNPKGNKKNAG
FTPQERQGFGELLQAVPLADSFRHLYPNTPYAYTFWTYMMNARSKNVGWRLDYFLLSHSLLPALCDSKIRSKALGSD
HCPITLYLAL

>p.S56QfsX22
MPKRGKKGAVAEDGDELRTEPEAKKSKTAAKKNDKEAAGEGPALYEDPPDQKTSPQWQTCHTQDLLLECGWASSLD

>p.S56VfsX26
MPKRGKKGAVAEDGDELRTEPEAKKSKTAAKKNDKEAAGEGPALYEDPPDQKTSPVANLPHSRSALGMWMGFEPGLR
RKD

S3 Fig. Sequence of the full-length APEX1 protein and its truncated variants. Sequences arising from a single-base insertion at coding position 165 (p.S56QfsX22) or a single-base deletion at coding position 165 (p.S56VfsX26) are shown. Changed polypeptide parts after the frameshift are highlighted red. The catalytic EEP domain is shown in magenta. The Cys65 residue critical for the Ref-1 activity is highlighted green.
