# Supplementary material for: Mild phenotype of knockouts of the major apurinic/apyrimidinic endonuclease APEX1 in a non-cancer human cell line
Source: PLoS One. 2021 Sep 16;16(9):e0257473. doi: 10.1371/journal.pone.0257473 (PMC8445474; doi:10.1371/journal.pone.0257473)
Supplement: S4 Fig — Mean ± s.d. are shown (n = 2–4; see Materials and methods for a detailed description)). None of the differences between knockout and wild-type cells were statistically significant (Student’s t rest). (PDF) [file pone.0257473.s005.pdf]

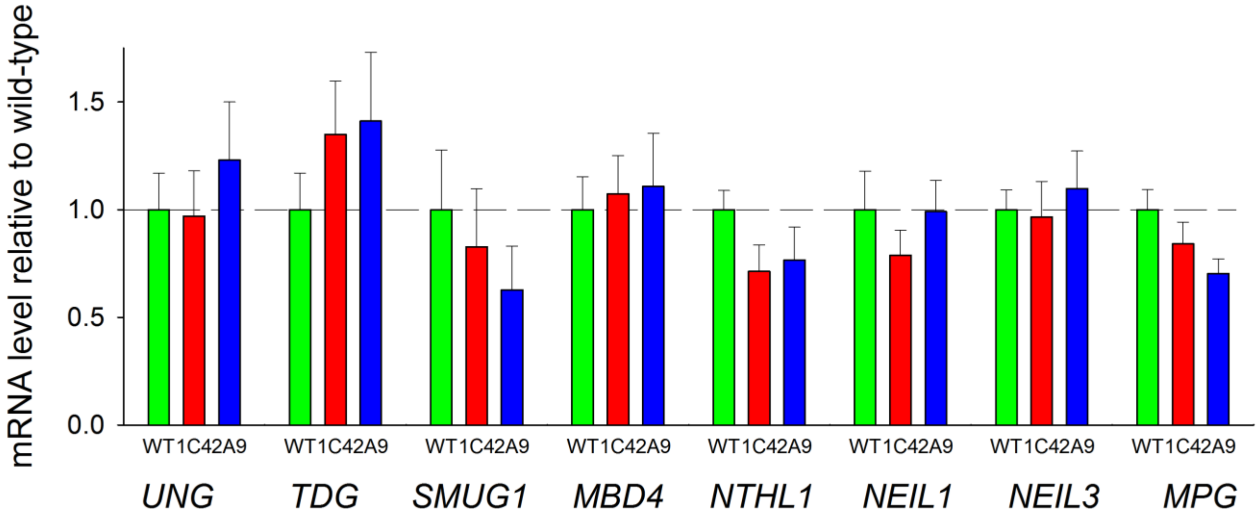

S4 Fig. mRNA levels of *UNG, TDG, SMUG1, MBD4, NTHL1, NEIL1, NEIL3,* and *MPG* genes coding for various DNA glycosylases in 1C4 and 2A9 cells relative to wild-type HEK 293FT. Mean ± s.d. are shown (n = 2–4; see Materials and Methods for a detailed description)). None of the differences between knockout and wild-type cells were statistically significant (Student's *t* rest).
