# Supplementary material for: Mild phenotype of knockouts of the major apurinic/apyrimidinic endonuclease APEX1 in a non-cancer human cell line
Source: PLoS One. 2021 Sep 16;16(9):e0257473. doi: 10.1371/journal.pone.0257473 (PMC8445474; doi:10.1371/journal.pone.0257473)
Supplement: S5 Fig — A, cleavage of a 23-mer duplex oligonucleotides containing an U:C pair by extracts of wild-type HEK293 (lane 3) and knockout cells 1C4 (lane 4) and 2A9 (lane 5). Lane 1, no enzyme or cell extract; lane 2, recombinant Ung. The reaction mixtures were treated with hot alkali to cleave DNA at AP sites formed by uracil removal. Arrows mark the mobility of the substrate (S) and the cleavage product (P). B, primer extension in a gapped substrate by extracts of wild-type HEK293 (lanes 3–4) and knockout cells 1C4 (lanes 5–6) and 2A9 (lanes 7–8). Lanes 1–2, recombinant POLβ. Arrows mark the mobility of the primer and the extension product. (PDF) [file pone.0257473.s006.pdf]

A

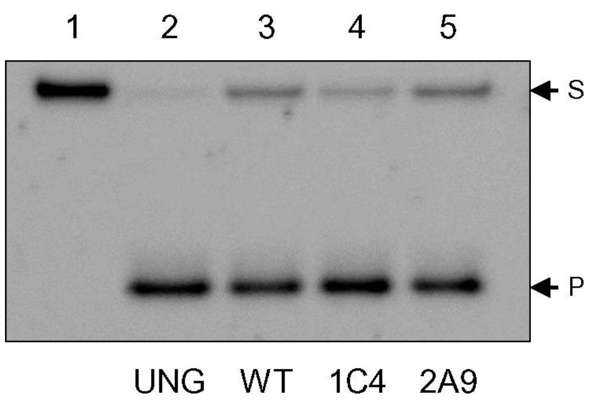

B

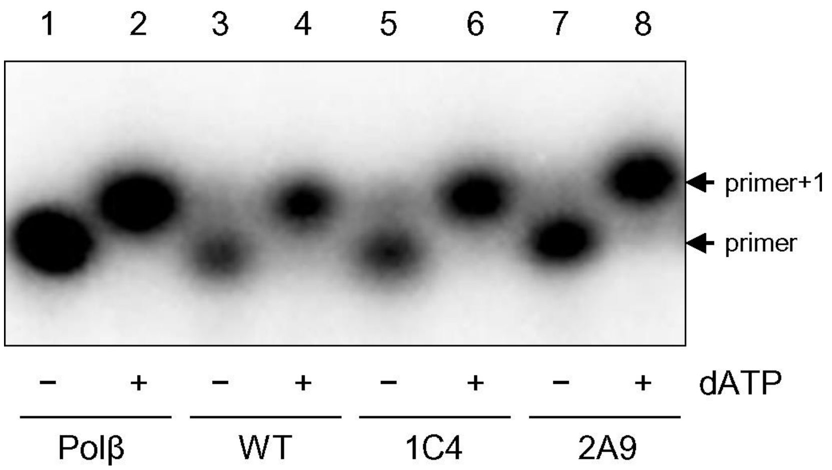

S5 Fig. Uracil–DNA glycosylase and gap-filling activities in cell extracts. A, cleavage of a 23-mer duplex oligonucleotides containing an U:C pair by extracts of wild-type HEK293 (*lane 3*) and knockout cells 1C4 (*lane 4*) and 2A9 (*lane 5*). *Lane 1*, no enzyme or cell extract; *lane 2*, recombinant Ung. The reaction mixtures were treated with hot alkali to cleave DNA at AP sites formed by uracil removal. Arrows mark the mobility of the substrate (S) and the cleavage product (P). **B,** primer extension in a gapped substrate by extracts of wild-type HEK293 (*lanes 3–4*) and knockout cells 1C4 (*lanes 5–6*) and 2A9 (*lanes 7–8*). *Lanes 1–2*, recombinant POLβ. Arrows mark the mobility of the primer and the extension product.
