# Supplementary figures and images for: Mild phenotype of knockouts of the major apurinic/apyrimidinic endonuclease APEX1 in a non-cancer human cell line
Source: PLoS One. 2021 Sep 16;16(9):e0257473. doi: 10.1371/journal.pone.0257473 (PMC8445474; doi:10.1371/journal.pone.0257473)

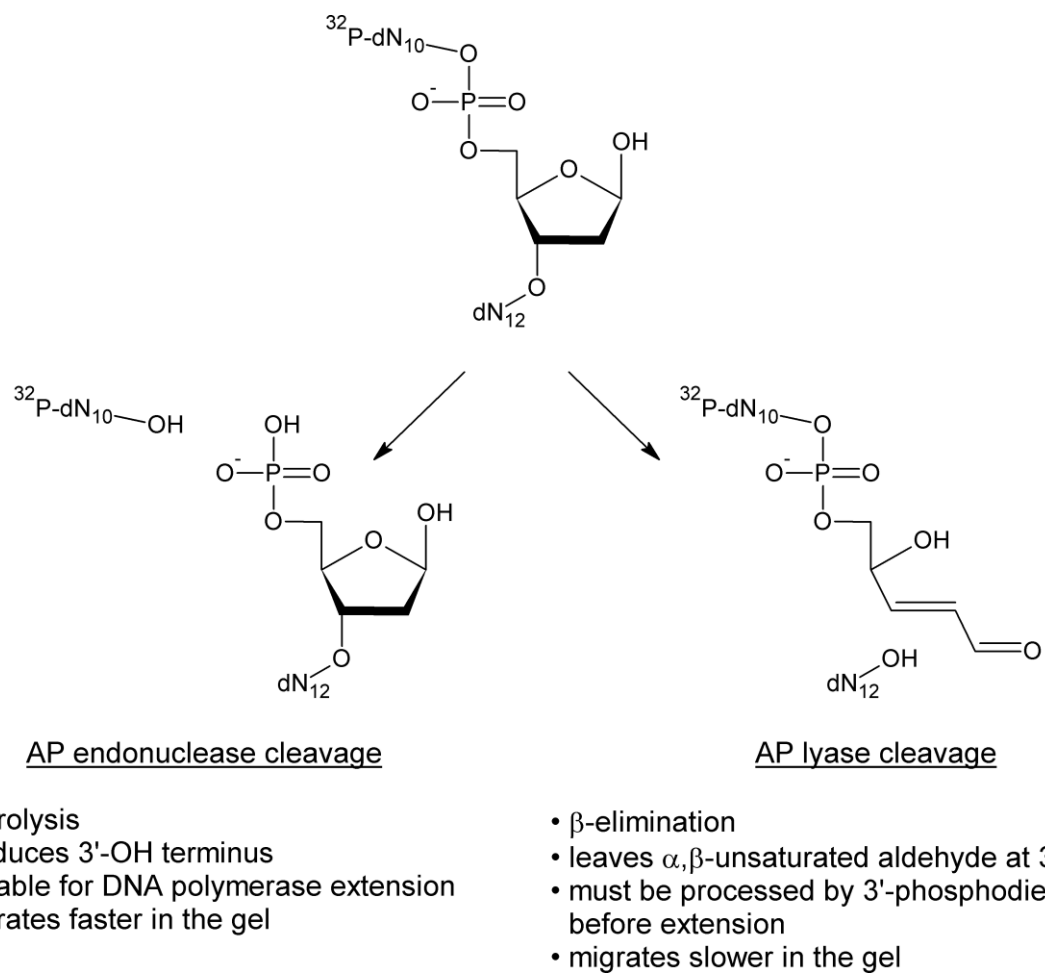

**S6 Fig. Products generated from AP site by AP endonucleases and AP lyases.**

Supplement: S6 Fig — (PDF) [file pone.0257473.s007.pdf]

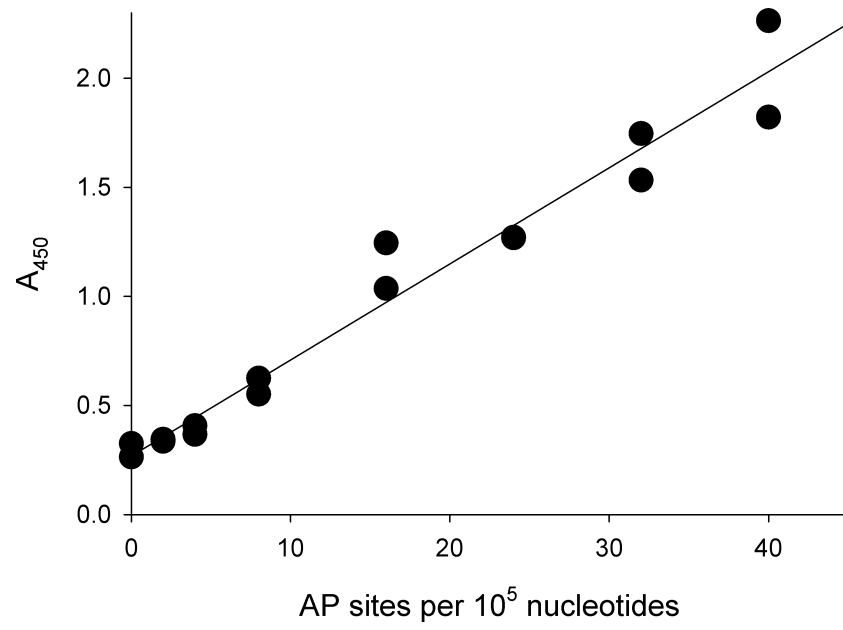

**S7 Fig. Calibration curve of the aldehyde reactive probe assay.**

Supplement: S7 Fig — (PDF) [file pone.0257473.s008.pdf]
