# Supplementary material for: Mild phenotype of knockouts of the major apurinic/apyrimidinic endonuclease APEX1 in a non-cancer human cell line
Source: PLoS One. 2021 Sep 16;16(9):e0257473. doi: 10.1371/journal.pone.0257473 (PMC8445474; doi:10.1371/journal.pone.0257473)
Supplement: S1 Raw images — (PDF) [file pone.0257473.s009.pdf]

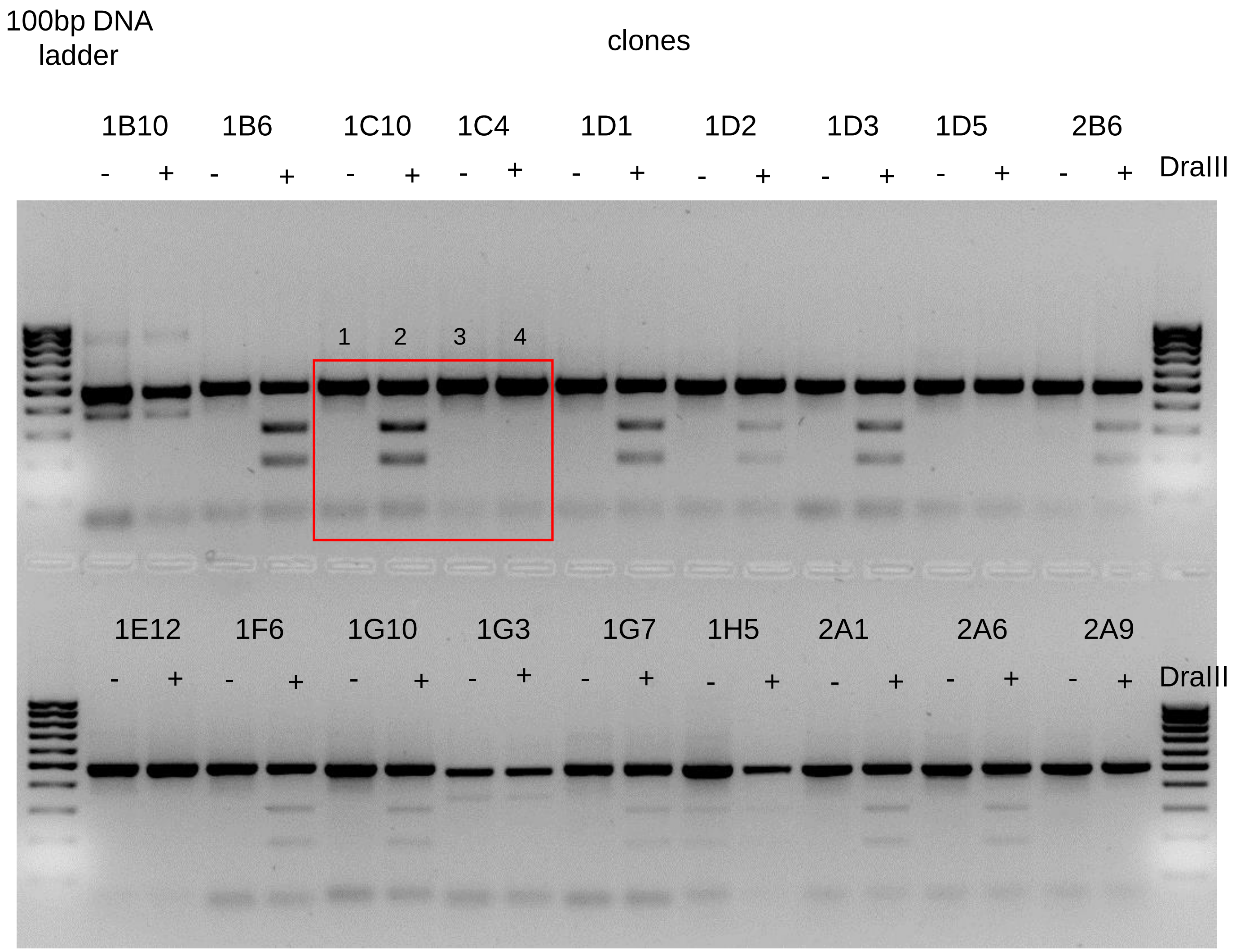

Original image for Fig 1C
Area included in Fig. 1C is boxed. Numbering of the lanes over the boxed area corresponds to that in Fig. 1C; detailed description can be found in the legend to Fig. 1C. The image was captured using a Vilber Lourmat Quantum gel documentation system.

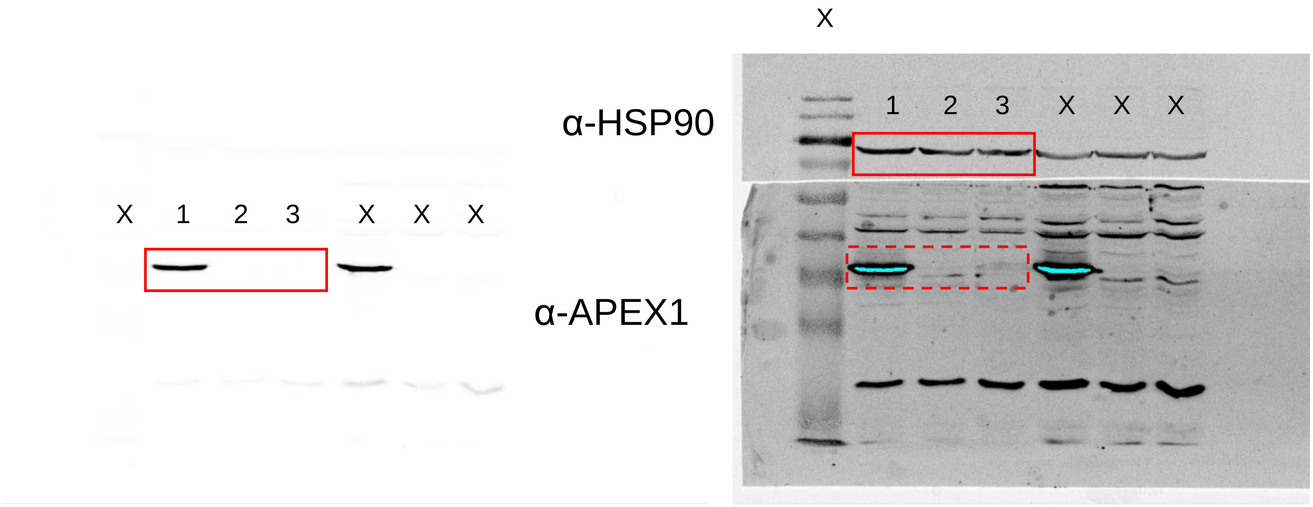

Original image for Fig 2A
Two imaged of the same membrane cut in two fragments: the uper part was stained for HSP90, the lower part, for APEX1. The images were acquired using an LI-COR Odyssey 9120 Imager with different laser intensity: left, low intensity to capture a non-saturated image after staining for APEX1; right, high intensity to capture an image after staining for HSP90. Areas included in Fig. 2A are boxed; the dashed box marks the oversaturated APEX1 bands in a high-intensity image (not included into Fig. 2A). Numbering of the lanes corresponds to that in Fig. 2A; detailed description can be found in the legend to Fig. 2A.

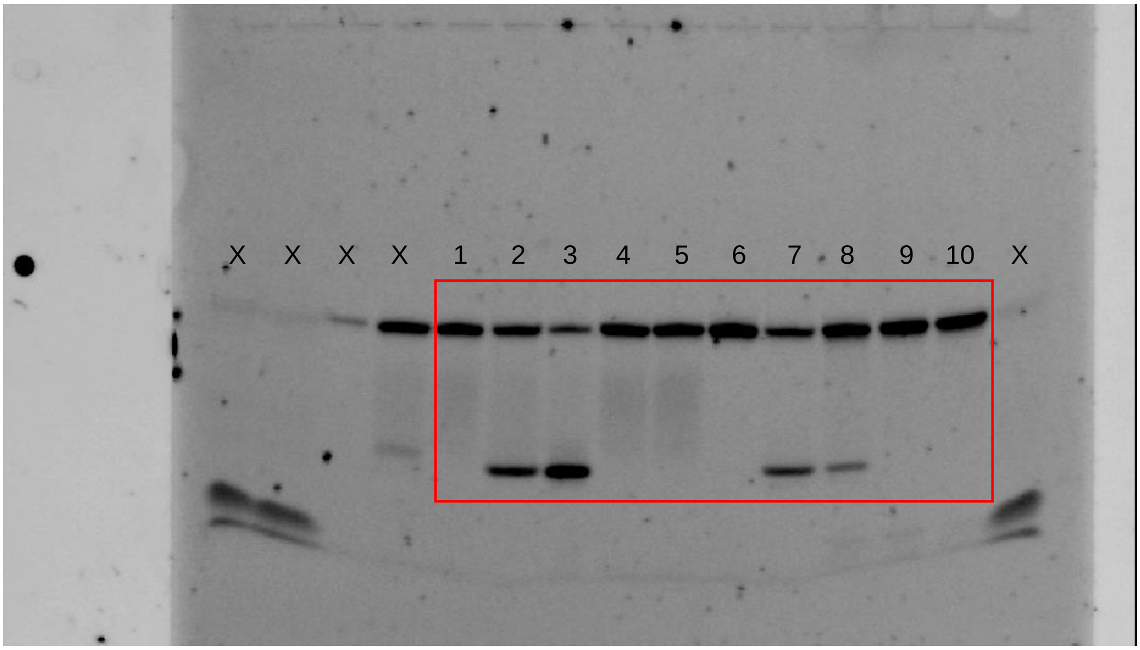

Original image for Fig 2C
Area included in Fig. 2C is boxed. Numbering of the lanes corresponds to that in Fig. 2C; detailed description can be found in the legend to Fig. 2C. The image was captured using a Typhoon FLA 9500 Imager in the fluorescent mode.

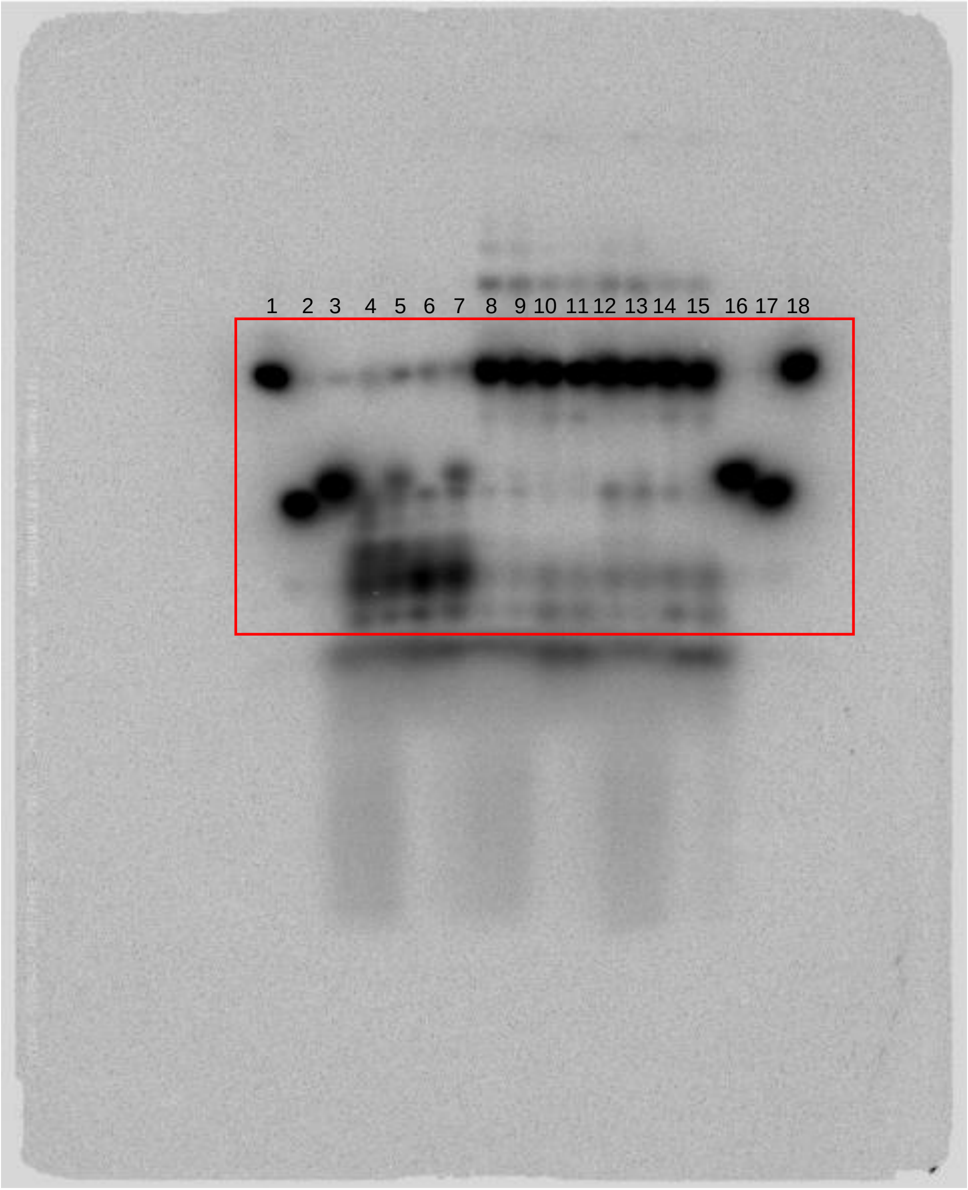

Original image for Fig 2D

Area included in Fig. 2D is boxed. Numbering of the lanes corresponds to that in Fig. 2D; detailed description can be found in the legend to Fig. 2D. The image was captured using a Typhoon FLA 9500 Imager in the phosphorimaging mode.

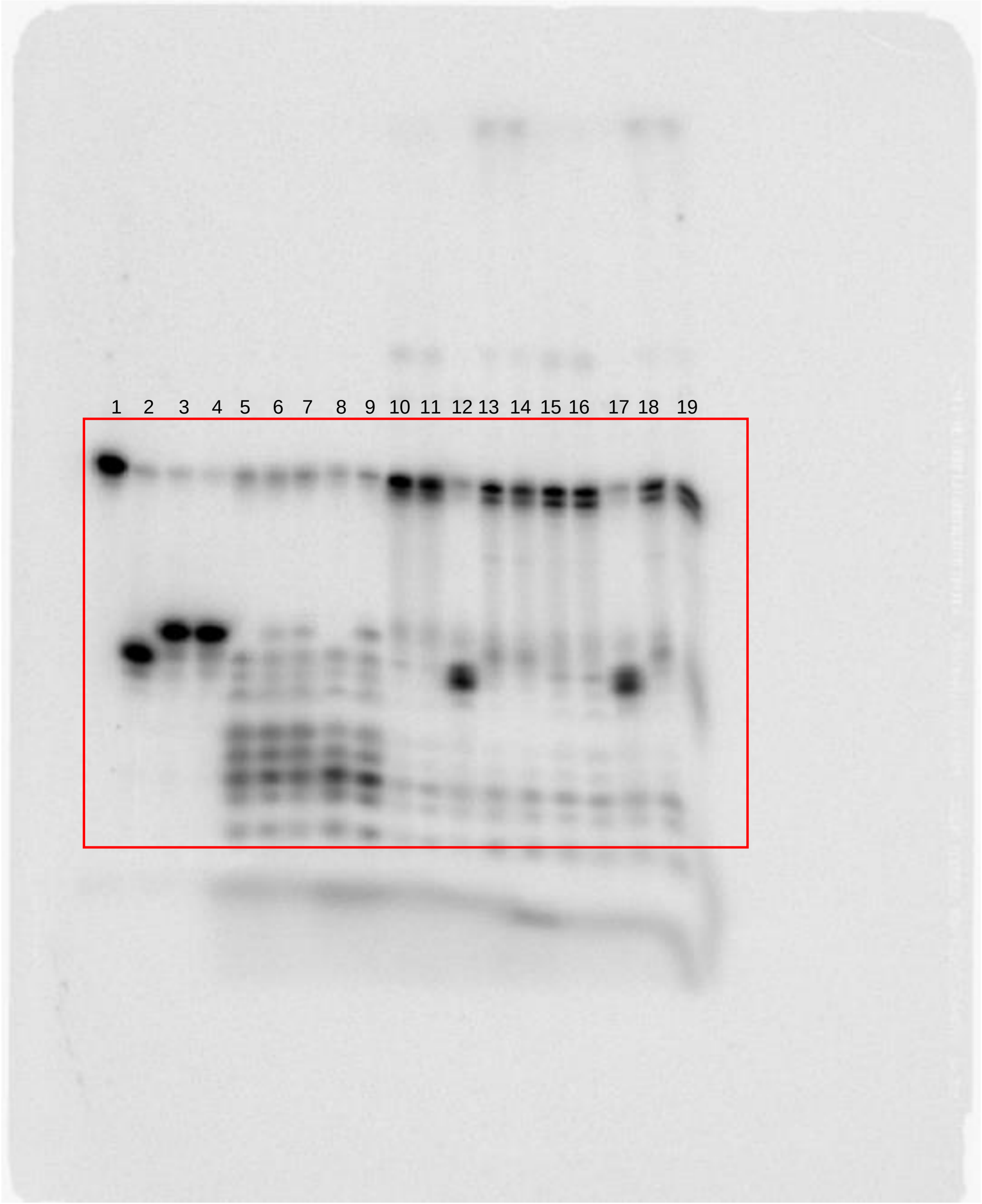

Original image for Fig 2E
Area included in Fig. 2E is boxed. Numbering of the lanes corresponds to that in Fig. 2E; detailed description can be found in the legend to Fig. 2E. The image was captured using a Typhoon FLA 9500 Imager in the phosphorimaging mode.

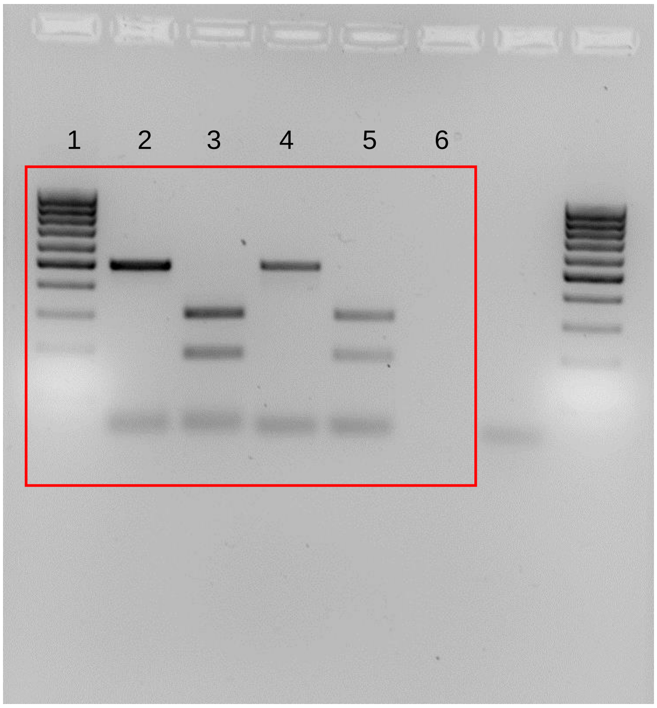

Original image for S2 Fig

Area included in S2 Fig is boxed. Numbering of the lanes over the boxed area corresponds to that in S2 Fig; detailed description can be found in the legend to S2 Fig. The image was captured using a Vilber Lourmat Quantum gel documentation system.

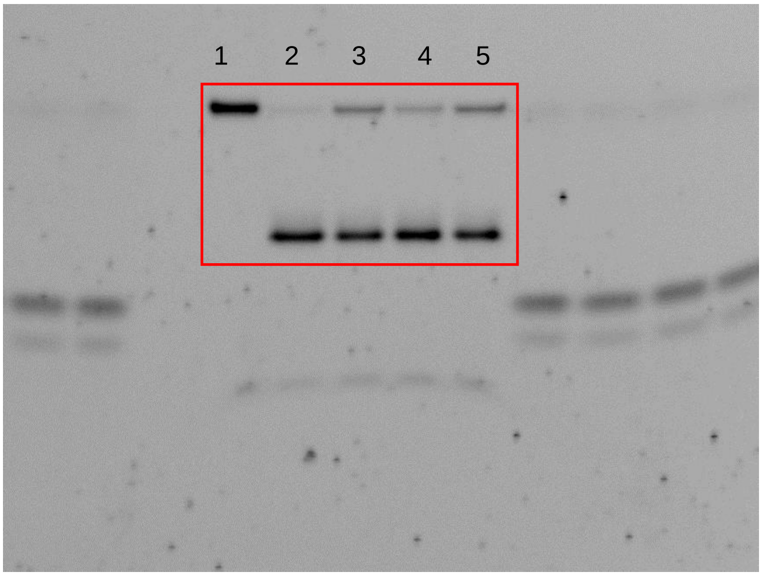

Original image for S5A Fig
Area included in S5A Fig is boxed. Numbering of the lanes over the boxed area corresponds to that in S5A Fig; detailed description can be found in the legend to S5A Fig. The image was captured using a Typhoon FLA 9500 Imager in the fluorescent mode.

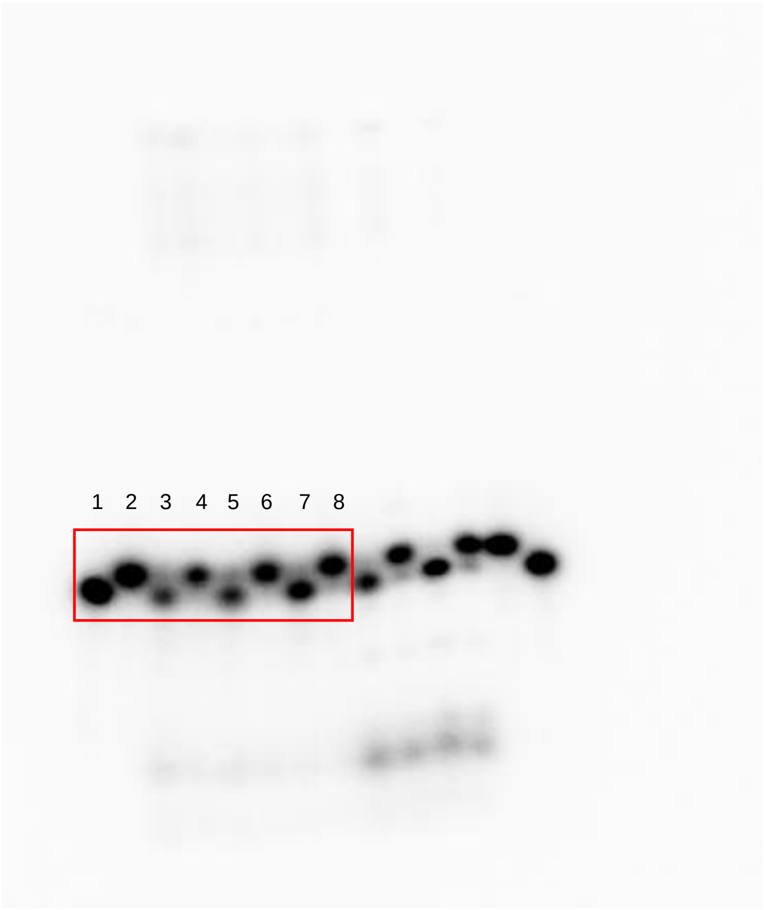

Original image for S5B Fig

Area included in S5B Fig is boxed. Numbering of the lanes over the boxed area corresponds to that in S5B Fig; detailed description can be found in the legend to S5B Fig. The image was captured using a Typhoon FLA 9500 Imager in the phosphorimaging mode.
